# Allocation of development assistance for health: is the predominance of national income justified?

**DOI:** 10.1093/heapol/czw173

**Published:** 2018-02-05

**Authors:** Olivier Sterck, Max Roser, Mthuli Ncube, Stefan Thewissen

**Affiliations:** 1 Department of Economics, and Oxford Department of International Development, University of Oxford, 3 Mansfield Rd, Oxford OX1 3TB, UK; 2 Institute for New Economic Thinking, Oxford Martin School, University of Oxford, Eagle House, Walton Well Rd, Oxford OX2 6ED UK; 3 Quantum Global Research Lab, and Blavatnik School of Government, University of Oxford; 4 Overseas Development Institute, Blackfriars Rd, London SE1 8NJ UK

**Keywords:** Communicable diseases, development assistance for health, gross national income, poverty

## Abstract

Gross national income (GNI) per capita is widely regarded as a key determinant of health outcomes. Major donors heavily rely on GNI per capita to allocate development assistance for health (DAH). This article questions this paradigm by analysing the determinants of health outcomes using cross-sectional data from 99 countries in 2012. We use disability-adjusted life years (Group I) per capita as our main indicator for health outcomes. We consider four primary variables: GNI per capita, institutional capacity, individual poverty and the epidemiological surroundings. Our empirical strategy has two innovations. First, we construct a health poverty line of 10.89 international-$ per day, which measures the minimum level of income an individual needs to have access to basic healthcare. Second, we take the contagious nature of communicable diseases into account, by estimating the extent to which the population health in neighbouring countries (the epidemiological surroundings) affects health outcomes. We apply a spatial two-stage least-squares model to mitigate the risks of reverse causality. Our model captures 92% of the variation in health outcomes. We emphasize four findings. First, GNI per capita is not a significant predictor of health outcomes once other factors are controlled for. Second, the poverty gap below the 10.89 health poverty line is a good measure of universal access to healthcare, as it explains 19% of deviation in health outcomes. Third, the epidemiological surroundings in which countries are embedded capture as much as 47% of deviation in health outcomes. Finally, institutional capacity explains 10% of deviation in health outcomes. Our empirical findings suggest that allocation frameworks for DAH should not only take into account national income, which remains an important indicator of countries’ financial capacity, but also individual poverty, governance and epidemiological surroundings to increase impact on health outcomes.

Key Messages

- Gross national income (GNI) per capita is not a significant predictor of the cross-country variation in health outcomes once other factors are taken into consideration.
- Instead, a country’s epidemiological surroundings, the poverty gap below a newly constructed health poverty line, and the institutional capacity are significant predictors of health outcomes.
- Our model captures 92% of the variation in health outcomes.
- This finding questions the paradigm of major donors to heavily rely on GNI per capita as an indicator for the allocation of development assistance for health.

## Introduction

Human potential lost to poor health is immense. In 2012, 39% of global potential healthy life years were lost to premature death or compromised by disability (Murray *et al.* 2015). There are large differences across regions, both in the total size of the disease burden and the type of prevailing diseases. Although in high-income countries 30% of potential healthy life years were lost, mostly because of non-communicable diseases (NCDs) and injuries (93%), as much as 74% of potential healthy life years were lost in Africa, two-thirds of which due to communicable diseases. The aim to end the epidemics of communicable diseases and to provide universal access to basic healthcare rose to the top of the global health agenda (Brolan and Hill 2015) and is now incorporated in the UN Sustainable Development Goals. To achieve this objective, Development Assistance for Health (DAH) has more than quintupled since 1990, to reach $36 billion per year in 2014 (Dieleman *et al.* 2015).

In order to improve global health outcomes, an important but controversial question is how DAH should be distributed across countries and over time. The present level of gross national income (GNI) per capita plays a key role in the eligibility criteria and allocation formulas of the nine largest multilateral organizations in terms of DAH funding size and geographical coverage (World Health Organization; the World Bank; Gavi; UNAIDS; UNICEF; UNDP; UNFPA; UNITAID; and the Global Fund; see Saxenian *et al.* 2015; Bump and Chi 2016). GNI per capita is generally complemented with other indicators, depending on the specific objective of the donor. GNI per capita is seen as a relatively simple and standardized proxy measure for a country’s level of development and its financial capacity to provide health services.

Does a higher level of national income improve the health of the population? Several articles have suggested this (Preston 1975; Pritchett and Summers 1996; Schell *et al.* 2007), but the evidence is ambiguous: The relationships between economic prosperity and mortality (Cutler *et al.* 2006; Deaton 2013) and childhood undernutrition (Vollmer *et al.* 2014) have been challenged. More generally, GNI per capita has been criticized as a measure of human development for not taking into account the inequality of incomes within countries and the access to health (Stiglitz *et al.* 2010; Farlow 2016). The nine previously mentioned largest multilateral organizations have acknowledged the importance of reflecting on the usage of GNI per capita as the sole measure of countries’ health needs and capacities by setting up the ‘Equitable Access Initiative’.

Is the level of GNI per capita a significant predictor of health outcomes across countries? Is the omnipresence of GNI per capita in the allocation of DAH justified by empirical evidence? This article is an empirical assessment of these questions.

**Figure 1.**
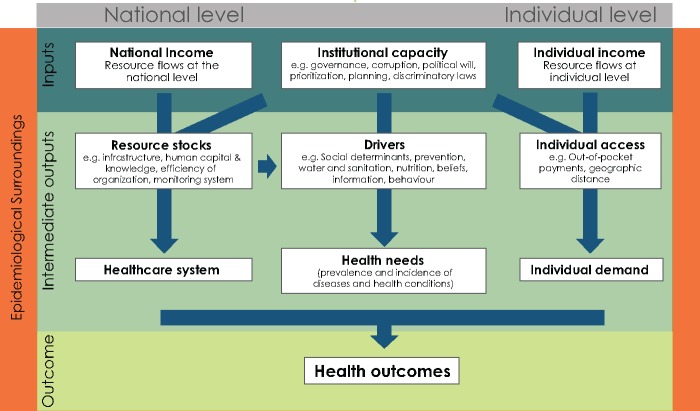
The health value chain within the epidemiological surroundings

Health outcomes are the result of a complex process involving economic, social, institutional and epidemiological constraints (Ataya *et al.* 2014). The health value chain in Figure 1 is a simplified representation of this process, by which inputs are turned into health outcomes in a country embedded in its international epidemiological surroundings.

The health value chain distinguishes three primary inputs: national income, institutional capacity and individual income. First, national income is an important factor for the domestic capacity to invest in health (Pritchett and Summers 1996; Anand and Bärnighausen 2004). Second, institutional capacity is pivotal for the construction of an efficient health system, for the prioritization of healthcare, for the design of policies affecting epidemiological and social determinants of disease burden, and for the implementation of legislation favoring individual access to health (Countdown Working Group on Health Policy and Health Systems 2008; Atun *et al.* 2013; Piot *et al.* 2015). Financial resources at the individual level are the third input. These affect whether individuals have sufficient income to purchase basic healthcare (Anand and Bärnighausen 2004).

The national health value chain is embedded in the country’s epidemiological surroundings, which we operationalize as the average disease burden in a country’s neighbouring countries. Many health problems are trans-boundary by nature, implying that the (lack of) actions of individual countries have consequences for their neighbours (Laxminarayan 2016). This particularly holds for poorer geographical areas, where the spread of diseases can lead to a ‘disease-driven poverty trap’ (Bonds *et al.* 2010) for multiple reasons. First, in poorer countries, a larger share of the disease burden is a consequence of infectious diseases that can spread more easily across borders. Second, outbreaks of new infections and spread of existing ones are accelerated by conditions that are present in large geographical parts of the developing world: rapid human population growth with land-use modifications, contact with wildlife (Jones *et al.* 2008), and population movement (Coker *et al.* 2004). Poor environmental conditions, water, sanitation, and malnutrition are main causes of diseases, including less contagious communicable, maternal, perinatal and nutritional conditions such as diarrheal episodes. Such environmental conditions can have trans-boundary consequences and can have common determinants, such as droughts or floods. More generally, infectious diseases make people more vulnerable to non-infectious communicable diseases and vice versa; overall poor health is contagious (Boutayeb 2006). Finally, infectious disease control is further limited by collective action problems for countries surrounded by fragile health systems and poor governance (Coker *et al.* 2004; Laxminarayan 2016).

The primary inputs determine the intermediate outputs of the health value chain. With institutional capacity, national income can be mobilized to generate necessary stocks of human and physical capital for healthcare infrastructure (Farlow 2016). Institutional capacity is also a major factor in affecting individual access to healthcare by determining factors such as out-of-pocket payments (Kumara and Samaratunge 2016) and the geographical distribution of health services (Ottersen *et al.* 2014). The available resource stock and the institutional capacity influence epidemiological and social determinants of health needs (Balabanova *et al.* 2013). These intermediate outputs affect the quality of the healthcare system, health needs in a society and individual demand for healthcare, ultimately determining the health outcomes of a country.

Our objective is to empirically assess whether the primary inputs and the epidemiological surroundings can explain the variation in health outcomes across countries. We first derive a health poverty line to measure individual income. After this we discuss our measures of the other inputs, the epidemiological surroundings and health outcomes. We then describe our identification strategy, present the results and discuss policy implications for the allocation of DAH.

## The 10.89 health poverty line

What is the minimum income a person needs to access basic healthcare? To answer this question, and set a health poverty line, we make use of the estimated costs of a basket of services and goods that are necessary to provide basic health services calculated by the Taskforce on Innovative International Financing for Health Systems (2009). These services include among others the cost of treating AIDS, TB and malaria, immunizations, treatment of acute respiratory infections, diarrheal diseases, maternal and perinatal conditions and malnutrition (for the entire list see Appendix 1 of the publication by the Taskforce). The Taskforce calculates the costs for this basket of health goods and services as an average across 49 low-income countries for the year 2005. The cost of the minimum health bundle in 2012 is 77.45 US$ or 198.73 international-$per person per year (see Appendix 1).

We make the assumption that individuals have the financial capacity to spend 5% of their income on health goods and services. This decision follows previous normative suggestions how much governments should spend on health as a percentage of their GDP (McIntyre and Meheus 2014). The World Health Report 2010 (WHO 2010) notes that ‘[…] those countries whose entire populations have access to a set of services usually have relatively high levels of [mandatory] pooled funds—in the order of 5–6% of gross domestic product’. There is evidence that households spend about 5% of their total expenditures on healthcare in low- and middle income countries (Makinen *et al.* 2000; Van Doorslaer *et al.* 2006).

Based on the costs of basic healthcare and the hypothesis that individuals have the financial capacity to spend 5% of their income on health, we derive a health poverty line of 3975 international-$per year or 10.89 international-$ per day (see Appendix 1). We express the poverty line in international-$to account for price differences across countries. We refer to this as the 10.89 health poverty line. Individuals whose income is below the 10.89 health poverty line are expected to face difficulties in obtaining access to basic healthcare services if universal healthcare is not provided. Therefore, the poverty gap, or the average shortfall of the total population from the 10.89 health poverty line, is a good indicator of the total lack of individual financial resources to finance healthcare within a country.

## Data sources

A list of all data sources can be found in Appendix 2, together with a table of descriptive statistics and correlation matrices.

### National income

Following the recommendation of Anand and Bärnighausen (2004), national income is measured by GNI per capita expressed in 2011 international-$sourced from the World Bank.

### Individual income

Data on the poverty gap come from the PovcalNet dataset published by the World Bank. These estimates are based on survey data from national statistical offices. The data do not account for in-kind public provision of healthcare and combine income and consumption information. We control for this in our sensitivity tests.

### Institutional capacity

Institutional capacity is measured by the Government Effectiveness Index published by the World Bank as part of the Worldwide Governance Indicators. This index maps perceptions of the quality of public services, the quality of the civil service and the degree of its independence from political pressures, the quality of policy formulation and implementation, and the credibility of the government's commitment to such policies. It is available for 215 countries on an annual basis since 1996.

For robustness checks we make use of the Control of Corruption Index, which is also published by the World Bank’s Worldwide Governance Indicators, and of the corruption perception index (CPI) sourced from Transparency International. For these three indicators, higher values indicate better governance.

### Health outcomes

We measure health outcomes by the disability-adjusted life years (DALYs) per 100 000 people. The DALYs are a standardized metric allowing for direct comparison and summing of burdens of different diseases. Data are available for all countries for 2000 and 2012. Conceptually, one DALY is the equivalent of 1 year in good health lost because of premature mortality or disability (Murray *et al.* 2015). Assessing health outcomes by both mortality and morbidity provides a more encompassing view on health outcomes than only looking at mortality or life expectancy alone.

**Figure 2.**
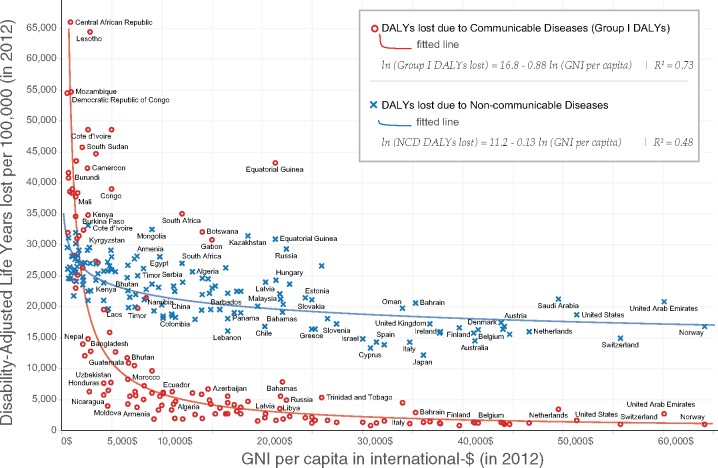
Scatter plots between DALYs lost per 100 000 and GNI per capita for 140 countries, distinguishing between Group I DALYs and NCD DALYs

Three categories of health conditions are distinguished: (1) Group I DALYs lost due to communicable, maternal, perinatal and nutritional conditions; (2) DALYs lost due to NCDs; and (3) DALYs lost due to injuries. Our main analysis focuses on Group I DALYs. This part of the burden of disease is by far the most important in our context. The DAH on Group I DALYs far exceeds the spending on other DALYs: whilst 49·8% of the total burden is associated with NCD DALYs, only 1·5% of all DAH is directed towards this latter category of diseases (Dieleman *et al.* 2015). Moreover, as Group I DALYs can be effectively controlled with a well-functioning health system, they provide the most useful measure to discriminate countries in terms of their health system effectiveness. Figure 2 confirms this by showing that log GNI per capita has a strong negative correlation with log Group I DALYs with an elasticity of −0·88, whereas log GNI per capita is not strongly related to log DALYs lost due to NCDs with an elasticity of −0·13. Another conclusion we can draw from Figure 2 is that the relationship between GNI per capita and DALYs lost due to the disease burden of Group I is best captured by a log-log function.

We assess the robustness of our regressions using under-5 mortality and maternal mortality as alternative dependent variables. One should however be cautious when using maternal mortality as cross-country data is sparse, and missing values are interpolated by the WHO, UNICEF, UNFPA, World Bank and UN (2015) using GDP per capita among other variables.

### Epidemiological surroundings

We measure the epidemiological surroundings of countries by a weighted average of the health outcomes (Group I DALYs lost) in neighbouring countries. Weights are given by the inverse of the Haversine distance between the centroids of countries. Similar results are obtained with an alternative spatial weighting matrix identifying countries sharing a common border (Appendix 3).

The Bayesian meta-regression technique employed to construct the estimates of the burden of disease presents a limitation of our analysis since the model uses, amongst many other sources of information, empirical data from surrounding countries. The WHO uses this method in descriptive epidemiology since in many parts of the world health data are sparse and the available data is of variable quality. To investigate this limitation we perform a robustness check in which we use child mortality data instead of data on the disease burden by the WHO. The estimates of child mortality rely on country-specific information only (You *et al.* 2015), and therefore should not induce a positive correlation between health outcomes across neighbouring countries. This robustness check confirms our main result.

## Methods

We use regression analysis to study the determinants of health outcomes. Since we are interested in explaining current health outcomes, we conduct a cross-sectional regression with data from 2012, which is the latest year for which data are available for all indicators.

We are interested in the primary drivers of health outcomes, as presented in the health value chain (Figure 1). In our main model, we therefore only include the three inputs and the epidemiological surroundings. Intermediate outputs in our value chain are excluded from the regression since these variables are output in our causal chain and are therefore ‘bad controls’ (Angrist and Pischke 2011). However, we test for the sensitivity of our results by including a number of often-mentioned intermediate variables. Our main equation is the following, with countries indexed by *i*:

$${\ln(Group\;I\;DALYs}_{i})=\;\beta_{0}+\beta_{1}{\;ln(GNI\;per\;capita}_{i})+\beta_{2}{\;poverty\;gap}_{i}+\beta_{3}\;{institutional\;capacity}_{i}+\beta_{4}{\;epidemiological\;\mathrm{surroundings}}_{i}+\varepsilon_{i}$$

We estimate this model for a set of poverty gap measures relative to a wide range of poverty lines, from the international poverty line of 1.90 international-$ per day, to our 10.89 health poverty line, up to 15 international-$ per day.

In order to avoid bias due to simultaneity between DALYs and DALYs in surrounding countries, we estimate a spatial lag regression model by generalized spatial two-stage least-squares (GS-2SLS) (Drukker *et al.* 2013). The weighting matrix is based on a country’s geographical coordinates.

Our benchmark model is specified in levels for two reasons. First, donors rely on current levels of GNI per capita to allocate DAH across countries. Second, Hausman (2001) underlines that ‘estimation of the fixed effects typically increases the variance of the noise relative to the variance of the signal’. Pischke (2007) explains that, in the presence of measurement errors, fixed effects and first difference estimations are ‘particularly worrisome when the measurement error is just serially uncorrelated noise, while the signal is highly correlated over time’. Our variables of interest are likely subject to measurement error (Kerner *et al.* 2015; Ferreira *et al.* 2016). The autocorrelation coefficients of our variables of interest are extremely high, ranging from 0.82 for the poverty gap based on the 1.90 international-$poverty line to 0.97 for GNI per capita (see Appendix 3 for correlation coefficients for years 2012 and 2000). Therefore, we prefer a cross-sectional regression to fixed-effects or first-difference estimation. Nevertheless, we will present results of fixed effects regressions as robustness check.

Even though we prefer a cross-sectional design to a panel design, it is important to acknowledge the limitations of this choice. With the cross-sectional design, regressions will be based a single point in time (2012) across 99 countries for which we have data. To lessen this concern, we will also show the results when using data from 2000. Another complication is the potential of reverse causality, as well as omitted variables bias and measurement error associated with national and individual income. To mitigate these risks, we follow Easterly (2007) and use the abundance of land suitable for growing wheat relative to that suitable for growing sugarcane as an instrument for the poverty gaps. Land endowments of sugarcane are suitable for commodities with economies of scale and slave labor, and are therefore historically associated with high inequality. Wheat is the premier land endowment example presenting opportunities for family firms and therefore stimulated the surge of a middle class. The F-test of a simple OLS regression of the poverty gap below the health poverty line on the instrument is equal to 16.91. We instrument domestic GNI per capita by a measure of average GNI per capita in neighbouring countries. Collier (2007) argues that countries with poor neighbours are in a development trap as the reduced sales market for their goods makes it harder to tap into world economic growth. The F-test of a simple OLS regression of GNI per capita on the GNI per capita of neighbouring countries is as high as 102.74, suggesting that the instrument is likely to be strong. Indicators of land endowment and GNI per capita in neighbouring countries are unlikely to be correlated with the residuals of the regression, implying that the exogeneity condition is likely to be satisfied.

The correlation between variables of interest is relatively high (correlation matrices are shown in Appendix 3), implying that regressions may be subject to multicollinearity. Multicollinearity does not bias the coefficients but increases their variance (Wooldridge 2015). We will therefore analyse the variance of coefficients associated with non-significant variables, to ensure that null results are not driven by multicollinearity. All variables are standardized to allow for comparison of relative effects across variables and regression models.^1^

## Results

Results of benchmark regressions are presented in Table 1. Column (1) presents the results of an OLS regression with only GNI per capita. Column (2) shows the result of an OLS regression for the 10.89 health poverty line. In Columns (3) to (10), we use GS-2SLS regressions and compare how results change when the poverty gap measure is based on different poverty lines. Column (11) displays the percentage contribution of each variable to deviation^2^ in health outcomes, based on Column (2) (Sterck 2016). This provides an indication of the size of the impact of each variable on health outcomes. We draw five conclusions from Table 1.

**Table 1.** OLS and GS-2SLS regressions to assess predictors of Group I DALY

	(1)	(2)	(3)	(4)	(5)	(6)	(7)	(8)	(9)	(10)	(11)
	Dependent variable: Group I DALYs lost per 100,000 (log)										
Poverty line	OLS		GS-2SLS								
(in international $)		10.89	1.9	3.1	5	7.5	10	10.89	12.5	15	Contrib.
GNI per capita (log)	−0.818*** (0.0585)	−0.0274 (0.0708)	−0.152** (0.0603)	−0.103 (0.0651)	−0.0260 (0.0698)	0.00204 (0.0712)	−0.0176 (0.0697)	−0.0274 (0.0690)	−0.0442 (0.0676)	−0.0645 (0.0657)	2%
Poverty gap *(see poverty line 1st row)*		0.256*** (0.0782)	0.0740 (0.0460)	0.141** (0.0592)	0.244*** (0.0730)	0.285*** (0.0794)	0.267*** (0.0786)	0.256*** (0.0775)	0.236*** (0.0753)	0.211*** (0.0721)	19%
Government effectiveness (WB)		−0.138*** (0.0406)	−0.150*** (0.0412)	−0.155*** (0.0407)	−0.156*** (0.0395)	−0.148*** (0.0390)	−0.140*** (0.0393)	−0.138*** (0.0395)	−0.135*** (0.0399)	−0.130*** (0.0404)	10%
Epidemiological surroundings		0.630*** (0.0496)	0.722*** (0.0494)	0.695*** (0.0511)	0.665*** (0.0521)	0.654*** (0.0532)	0.658*** (0.0538)	0.662*** (0.0537)	0.669*** (0.0535)	0.679*** (0.0529)	47%
Constant	4.28e-09 (0.0582)	1.71e-09 (0.0284)	−0.0111 (0.0289)	−0.0107 (0.0285)	−0.0102 (0.0277)	−0.0101 (0.0274)	−0.0101 (0.0276)	−0.0102 (0.0277)	−0.0103 (0.0278)	−0.0105 (0.0280)	21%
Observations	99	99	99	99	99	99	99	99	99	99	
*R*^2^	0.668	0.924									

Standard errors in parentheses.

* *P* < 0.10, **P < 0.05, ***P < 0.01. The sample includes 99 countries for which all data is available (see Appendix 1). All variables are standardized.

First, GNI per capita (in log) is significantly correlated with Group I DALYs (in log) when other factors are omitted (Column (1)), or when the poverty gap measure is based on a very low poverty line (Column (3)). However, the coefficient associated with GNI per capita (in log) decreases and becomes insignificant when the poverty line approaches the 10.89 health poverty line. This null result is not driven by multicollinearity. The standard deviation of the coefficient associated with GNI per capita only marginally increases when other variables are included in the model. In the OLS regression presented in Column (2), the Variance Inflation Factor of the poverty gap measure and GNI per capita (log) are well below the rule of thumb of 10 signaling serious multicollinearity (7.52 and 6.16, respectively) (O’Brien 2007).

Second, the poverty gap measures are highly significant across all poverty lines. The largest coefficient is reported for the 7.5 international-$poverty line, but this coefficient does not statistically differ from the coefficient of the 10.89 health poverty line. For the 10.89 health poverty line, the poverty gap captures 19% of deviation in health outcomes (log). A one-standard-deviation increase in the poverty gap (25% points) increases the predicted DALYs lost due to Group I diseases per life year by 29%.

Third, the coefficients of the Government Effectiveness Index are statistically different from zero across all specifications, showing that institutional capacity is an important factor of access to health. This variable captures 10% of deviation in health outcomes (log).

Fourth, we find that coefficients measuring the strength of spatial correlation are positive and highly significant across all specifications. The epidemiological surroundings in which countries are embedded account for 47% of the standard deviation in health outcomes (log) in our preferred model with the 10.89 health poverty line. This demonstrates that the epidemiological surroundings are the most important factor explaining health outcomes.

Finally, we emphasize that the fit of the regressions is very high. There is no easily interpretable measure of goodness of fit for our preferred GS-2SLS estimation. However, Column (2) shows that the *R*^2^ of the OLS estimation is as high as 0.92. As we show in Appendix 3, the Wald Chi-squared statistics of the maximum likelihood estimation is also extremely high (*X*^2^ = 93.9, *P*-value = 3.13 10e-20). The primary inputs identified in the health value chain explain almost all variation in health outcomes.

We assess the robustness of results in Table 2. Column (1) shows the results of our benchmark regression, in which the dependent variable is the log of Group I DALYs. In Column (2), we demonstrate the robustness of our results when the abundance of land suitable for growing wheat relative to that suitable for growing sugarcane is used as an instrument for the poverty gaps, and when GNI per capita is instrumented by a measure of GNI per capita in neighbouring countries.

**Table 2.** Robustness regressions—alternative dependent variables

	(1)	(2)	(3)	(4)	(5)	(6)	(7)	(8)	(9)
	DALYs from WHO						DALYs	Mortality	
Dependent variables	Group I					NCDs	IHME	U5	Maternal
Method/year	Original	IV	2012	2000	Panel		2013		
GNI per capita (log)	−0.0274 (0.0690)	0.516 (0.341)	−0.0186 (0.0802)	−0.0795 (0.0886)	−0.192 (0.141)	−0.0646 (0.186)	−0.0470 (0.0721)	0.0260 (0.0822)	−0.0806 (0.0868)
Poverty gap (*10.89 international-$*)	0.256*** (0.0775)	1.030** (0.459)	0.274*** (0.0872)	0.227** (0.0920)	−0.0490 (0.139)	0.140 (0.184)	0.305*** (0.0802)	0.436*** (0.0888)	0.368*** (0.0934)
Government effectiveness (WB)	−0.138*** (0.0395)	−0.169** (0.0753)	−0.133*** (0.0465)	−0.0363 (0.0494)	−0.0255 (0.114)	−0.271** (0.106)	−0.108*** (0.0413)	−0.206*** (0.0470)	−0.113** (0.0496)
Epidemiological surroundings	0.662*** (0.0537)	0.393** (0.186)	0.658*** (0.0616)	0.718*** (0.0657)	0.885** (0.344)	0.377 (0.234)	0.615*** (0.0557)	0.478*** (0.0647)	0.502*** (0.0692)
Constant	−0.0102 (0.0277)	−0.00392 (0.0462)	−0.0220 (0.0331)	−0.0245 (0.0369)	0.0133 (0.103)	−0.00757 (0.0741)	−0.00967 (0.0289)	−0.00702 (0.0330)	−0.00633 (0.0348)
Observations	99	78	84	84	84	99	99	99	99

Standard errors in parentheses.

* *P* < 0.10, **P < 0.05, ***P < 0.01. All variables are standardized.

In Columns (3) and (4), we assess how the relationship between Group I DALYs and primary inputs evolved over time using data for 2000 and 2012 while keeping the sample constant. Both the health poverty gap and the epidemiological surroundings are strong predictors of health outcomes in 2000 and 2012. National income on the contrary is not predicting health outcomes in either 2000 or 2012. The coefficient associated with institutional capacity is not significant with 2000 data. Overall, we conclude that the relationship is relatively stable over time.

In Column (5), we look at within-country variation by applying a fixed effect model with 2000 and 2012 data. As emphasized in ‘Methods’ section, this approach is expected to suffer from attenuation bias due to measurement errors and high autocorrelation in variables of interest. The reduced number of countries with data available for both years is expected to further increase standard errors. Only the epidemiological surroundings appear to be an important driver of the burden of disease with this specification. The coefficient associated with GNI per capita is negative, but not significant at conventional levels (*P*-value = 0.173). The coefficients of the poverty gap and government effectiveness indicators are close to zero and not significant.

In Column (6) we apply the same specification to DALYs from NCDs. Results are in accordance with Figure 2: the predictors of NCDs are very different from those of communicable, maternal, perinatal and nutritional conditions. National income, the poverty gap, and the epidemiological surroundings do not significantly predict the burden of NCDs. The fact that the epidemiological surroundings are only significant for Group I DALYs lost and not for NCDs points to the contagious nature of the diseases of Group I DALYs lost and its trans-boundary determinants and consequences. Only the measure of institutional capacity is a significant predictor. GNI per capita and the poverty gap are also not significantly correlated with the DALYs lost due to injuries (results not shown here). These findings suggest that understanding the predictors of NCDs and injuries requires further analysis, which is beyond the purview of this paper. In Column (7), we show that results are robust when we use Group I DALYs lost as measured by the Institute for Health Metrics and Evaluation (IHME) (Murray *et al*. 2015). This is not surprising given the high correlation between the indicators constructed by WHO and by the IHME (Appendix 3).

In Columns (8) and (9), we show that our results are robust if we consider two alternative indicators of health outcomes widely used in the literature: under-five mortality and maternal mortality. The significant predictors are the same as in the model specification with Group I DALYs.

In Table 3, we further assess the robustness of our results by changing the specification of the estimated model. The results are not significantly affected when alternative measures of institutional capacity are considered (Columns 1–3). The results are also robust to the inclusion of a dummy indicating whether poverty data from Povcal is based on income or consumption data (Column 4). Similarly, results are unchanged if we control for other predictors of health outcomes mentioned in the literature, such as the share of out-of-pocket expenditures in total expenditures for health (Column 5; e.g. Kumara and Samaratunge 2016), for education (Column 6; Pritchett and Summers 1996; Gakidou *et al.* 2010), or for fertility (Column 7; Makepeace and Pal 2008). Even though the coefficient associated with fertility rate is positive and significant, we do not include this variable in the main regression because of the high risk of reverse causality associated with this variable (e.g. Preston 1978). Other coefficients are not significantly affected by the inclusion or removal of this variable. Results are also similar if we control for regional dummies (Column 8).

**Table 3.** Robustness checks with alternative independent variables

	(1)	(2)	(3)	(4)	(5)	(6)	(7)	(8)
	Dependent variable: Group I DALYs lost per 100,000 (log)							
GNI per capita (log)	−0.0274 (0.0690)	−0.0732 (0.0666)	−0.0825 (0.0677)	−0.0273 (0.0689)	−0.0173 (0.0704)	−0.00670 (0.0776)	0.0253 (0.0674)	−0.00237 (0.0701)
Poverty gap (*10.89 international-$*)	0.256*** (0.0775)	0.250*** (0.0785)	0.249*** (0.0805)	0.254*** (0.0777)	0.266*** (0.0790)	0.345*** (0.0882)	0.233*** (0.0730)	0.254*** (0.0789)
Government effectiveness (WB)	−0.138*** (0.0395)			−0.140*** (0.0397)	−0.152*** (0.0440)	−0.127*** (0.0449)	−0.117*** (0.0381)	−0.150*** (0.0396)
Control of Corruption (WB)		−0.101*** (0.0330)						
CPI 2012 Score			−0.0856** (0.0334)					
Povcal type dummy				0.0264 (0.0701)				
Out-of-pocket payments					−0.0225 (0.0326)			
Mean Year of Schooling						0.00143 (0.0565)		
Fertility rate (log)							0.214*** (0.0649)	
Asia dummy								−0.172 (0.134)
Europe dummy								−0.178 (0.214)
North America dummy								−0.180 (0.152)
South America dummy								−0.200 (0.174)
Epidemiological	0.662***	0.670***	0.673***	0.670***	0.656***	0.609***	0.538***	0.606***
surroundings	(0.0537)	(0.0542)	(0.0554)	(0.0532)	(0.0544)	(0.0574)	(0.0622)	(0.0900)
Constant	−0.0102 (0.0277)	−0.0103 (0.0280)	−0.0102 (0.0286)	−0.0178 (0.0341)	−0.0101 (0.0276)	−0.0108 (0.0290)	−0.00829 (0.0263)	0.108 (0.108)
Observations	99	99	98	99	99	86	99	99

Standard errors in parentheses.

* *P* < 0.10, **P < 0.05, ***P < 0.01. All variables are standardized.

In Appendix 3, we further show that our results remain valid when using maximum likelihood estimators, or when we apply an alternative spatial weighting matrix using neighbouring borders instead of geographical coordinates. In Appendix 4, we show that results are not significantly affected when we control for geographical variables (absolute latitude, terrain ruggedness, soil quality, tropical climate, distance to coast and average temperature), for variables capturing population characteristics (population size in log, population density, urban share, migrant share and ethnolinguistic fragmentation and polarization), for the intensity of conflict (number of deaths in log) and the intensity of natural disasters (number of deaths in log and number of people affected in log), as well as for all these variables in surrounding countries. Results remain qualitatively similar when we control for GNI per capita (log) in surrounding countries, for the poverty gap in surrounding countries, and for government effectiveness in surrounding countries; these latter estimates should however be interpreted with caution because of high multicollinearity. The coefficients of the poverty gap and of government effectiveness remain highly significant when the epidemiological surroundings are removed from the list of controls, while GNI per capita (log) remains insignificant.

## Conclusion

In this article, we provide evidence suggesting that the level of GNI per capita is not a significant predictor of health outcomes as measured by DALYs lost due to Group I diseases in 2012 for 99 low- and middle-income countries once other factors are properly accounted for. Our analysis contributes to the literature on health outcomes and universal health coverage by demonstrating the importance of the epidemiological surroundings, individual poverty, and the institutional capacity for explaining the cross-country variation in Group I DALYs.

We make two technical contributions. First, we calculate that 10.89 international-$ per day is the level of income that an individual needs to finance basic healthcare when free and universal healthcare coverage is lacking. This level of income can be used as a health poverty line. The poverty gap at the country level below this poverty line explains 19% of the deviation in Group I DALYs. Our second technical contribution is the incorporation of the epidemiological surroundings of countries, which are shown to be the most important factor of health outcomes. We correct for reverse causality using a spatial two-stage least-squares estimation technique.

Our paper contributes to recent initiatives that reflect on allocation formulas for DAH to better track health needs and capacities of countries. On the one hand, GNI per capita is a good measure of countries’ financial capacities, and as such, this indicator should play an important role in allocation formulas. Inclusive growth also reduces poverty, and lower levels of poverty reduce the burden of disease. On the other hand, however, our empirical evidence suggests that GNI per capita does not seem to be a direct determinant of health outcomes. To be sure, GNI per capita is a reasonably good proxy for explaining health outcomes: it is highly correlated to health outcomes and explains 67% of the cross-country variation in health outcomes if other factors are ignored. Still, this leaves a substantial part of the disease burden unexplained. Moreover, GNI per capita is no longer significant when other factors are taken into consideration. The importance of individual poverty in explaining health outcomes shows that the level of national income fails to reflect the degree of universal healthcare coverage and the inclusiveness of total income in a country (Stiglitz *et al.* 2010; Watkins 2014). The level of national income also does not provide information on the potentially contagious diseases and their trans-boundary determinants and consequences. Furthermore, national income does not take stock of government effectiveness or institutional capacity. More generally, in the short run, countries might not have time to materialize their domestic resources and generate a well-functioning universal healthcare system.

**Figure 3.**
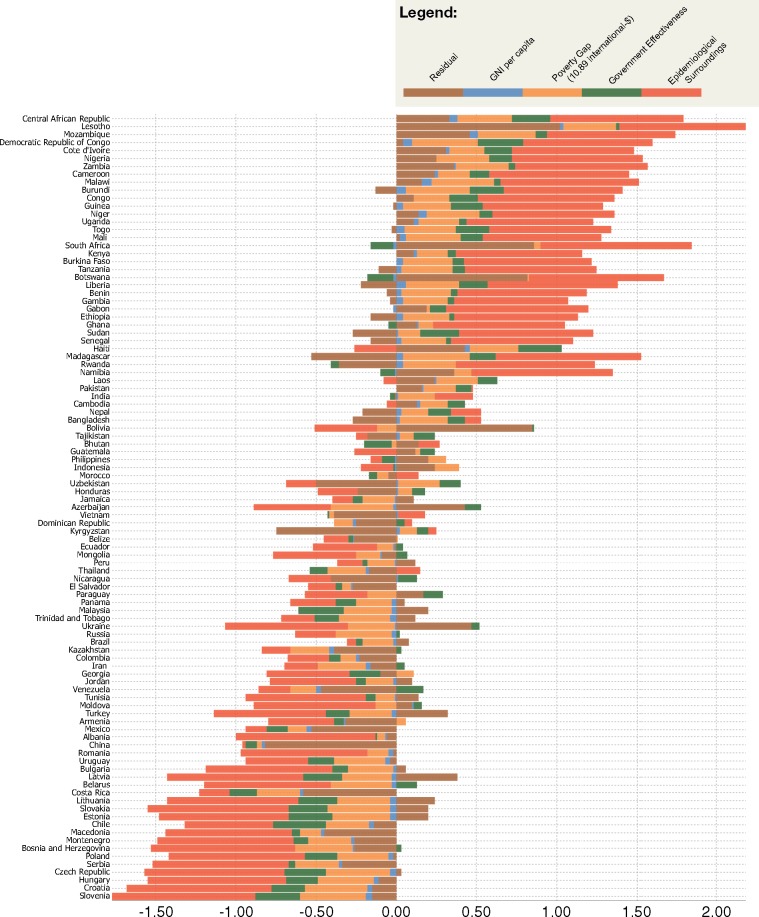
Decomposition of the contributions of our independent variables on Group I DALYs per country

For normative and efficiency reasons, donors may want to unpack the underlying factors of countries’ health outcomes, as shown in Figure 3, and take these into account when allocating DAH. Donors may want to particularly compensate countries that are highly vulnerable to the epidemiological surroundings, for three reasons. First, it might be argued that countries are not directly responsible for a large share of their disease burden, as part of it is the result of contagious communicable diseases in neighbouring countries. Second, given the large externalities, donors could focus their spending on supranational regions with large shares of contagious communicable diseases for efficiency reasons. Third, vulnerability to the epidemiological surroundings may create a ‘health trap’ and a ‘poverty trap’, where countries are stuck in a situation of poor health and poverty due to their geographical location (Bonds *et al.* 2010).

Donors may weigh poverty and the institutional capacity in their allocation of DAH in multiple ways depending on their priorities. DAH in the short run may be increased in countries with high levels of poverty, to reach poorer citizens suffering from a lack of health access. On the other hand, giving more aid to countries with high levels of poverty comes with disincentives. Instead, donors may want to reduce DAH to foster redistributive and inclusive change, or impose conditionalities on aid. Comparable arguments can be made for institutional capacity.

Given the complexity of health and allocation of DAH, this study has several limitations. First, our conclusions are restricted to Group I DALYs. NCDs, which tend to become a larger share of a country’s burden of disease when it develops, are different in nature, as we showed in our regressions (Atun *et al.* 2013; Sacco *et al.* 2013). Individual poverty and the epidemiological surroundings do not explain variation in this latter group of diseases.

Second, limited data availability implies that our benchmark regressions are based on cross-sectional data for 99 countries. Although our main results hold for a large set of sensitivity tests, one should remain cautious when interpreting our results and utilizing them for policy-making. Future research should improve our estimates when better data becomes available.

Third, we deliberately restricted our empirical analyses to the primary inputs and the epidemiological surroundings. Factors such as the healthcare infrastructure, social determinants, or human capital are intermediate outputs in our causal chain and are therefore ‘bad controls’ (Angrist and Pischke 2011). Identifying country-specific deficiencies in intermediate outcomes becomes important when deciding how health budgets should be spent at the national level.

Finally, further study should be devoted to improving current allocation systems. Given that constructing such an allocation formula requires complex normative position-taking, we refrain from doing so directly, but we hope that our study provides tools and insights for getting there.

## Supplementary Data

Supplementary data are available at *HEAPOL* online.

## Funding

This work was supported by the Wellcome Trust [099114/Z/12/Z].

*Conflict of interest statement*. None declared.

## Supplementary Material

Supplementary Appendix

## Footnotes

1 To standardize variables, we subtract their mean and divide the result by their standard deviation.

2 The percentage contribution of each variable refers to contribution to the standard deviation (and not to the variance) of the dependent variable, implying the sum of contributions does not add up to the *R*^2^.
