# Supplementary material for: Allocation of development assistance for health: is the predominance of national income justified?
Source: Health Policy Plan. 2018 Feb 5;33(Suppl 1):i14–23. doi: 10.1093/heapol/czw173 (PMC5886300; doi:10.1093/heapol/czw173)
Supplement: Supplementary Appendix [file czw173_hpp_appendix_revision.docx]

**Appendix 1 - Deriving the 10.89 health poverty line**

Taskforce on Innovative International Financing for Health Systems (2009) calculated a price for a *minimum bundle of health goods and services* of 54 US-$. This is an average over 49 poor countries and this consumption bundle has a non-traded part (62%) and a traded part (the remaining 38%).

We convert this into 2012 US-$ by taking the US-inflation into account to adjust for price changes of the imported goods and by separately taking inflation in the 49 poor countries into account to adjust for the price changes of the domestically purchased part of the health bundle.

We take the price change in the 49 poor countries from Meheus and McIntyre (2014) who report a 59% price increase between 2005 and 2012. We take the price change in the US from the OECD which report an 18% price increase (CPI) between 2005 and 2012.

$$\left( 54 US\$\times0.38 \right)\times\left( 1+0.18 \right)+\left( 54 US\$ \times0.62 \right)\times\left( 1+0.59 \right)= 77.45 US\$ (in 2012 prices)$$

We then calculate the population weighted average price level ratio of PPP conversion factor (GDP) to market exchange rate for the benchmark year of the latest ICP, which was 2011. The data for this are taken from the World Bank’s World Development Indicators. For the 49 countries this ratio relative to the US is 0.39.

$$\frac{77.45 US\$}{0.39}=198.73 international \$$$

We use this price of the *minimum bundle of health goods and services* in international $ in 2012 as the starting point for the calculation of an international health poverty line. As explained above, we suggest that individuals should have the financial capacity to spend 5% of their income on health goods and services: this means the international poverty line in 2012 is 198.73 international-$*20 = 3974.62 international-$ (in 2011 prices).

The international health poverty line is therefore:

- 3974.62 international-$ per year
- 10.89 international-$ per day

**Data used:**

– The "High Level Taskforce for Innovative International Financing of Health Systems” (<http://bit.ly/1SIDN3f>) calculated a price for a *minimum bundle of health goods and services* of 54 US-$. This is an average over 49 poor countries.

- For 2011 the population weighted “Price level ratio of PPP conversion factor (GDP) to market exchange rate“ is .38970678 (rounded to 0.39)
  - <http://data.worldbank.org/indicator/PA.NUS.PPPC.RF>
  - <http://data.worldbank.org/indicator/SP.POP.TOTL>

**Appendix 2 – Data sources and descriptive statistics**

| Variable | Source |
| --- | --- |
| DALYs | WHO (2016) – Global Health Observatory data repository. Disability-adjusted life years (DALYs), 2000-2012 (Accessed 28^th^ January 2016).  <http://apps.who.int/gho/data/node.main.DALYRATEWORLD?lang=en>  Group I DALYs is the disease burden related to “Communicable, maternal, perinatal and nutritional conditions ”. We have calculated the disease burden that is only related to communicable disease burden by restricting it to the two subgroups of Group I which are called “infectious and parasitic diseases” and “respiratory infections”. |
| Maternal mortality | WHO, UNICEF, UNFPA, World Bank Group and the United Nations Population Division (2015) – Trends in maternal mortality: 1990 to 2015. WHO /RHR/15.23 (Accessed 25^th^ October 2016).  <http://data.unicef.org/topic/maternal-health/maternal-mortality/> |
| Under-5 mortality | WHO, UNICEF, UNFPA, World Bank Group and the United Nations Population Division (2015) – Trends in maternal mortality: 1990 to 2015. WHO /RHR/15.23 (Accessed 26^th^ February 2016).  http://apps.who.int/gho/data/node.main.ChildMort-1?lang=en |
| GNI per capita | The GNI per capita expressed in 2011 international-$ sourced from the World Bank (accessed January 6^th^, 2016). |
| Poverty data | Data on the poverty gap come from the PovcalNet dataset published by the World Bank (accessed 3^rd^ March 2016).  For a recent discussion, see FERREIRA, Francisco HG, CHEN, Shaohua, DIKHANOV, Yuri, *et al.* A global count of the extreme poor in 2012: data issues, methodology and initial results. *World Bank Policy Research Working Paper*, 2015, no 7432 |
| Government Effectiveness and corruption | World Bank WGI data (accessed February 14^th^, 2015).  http://info.worldbank.org/governance/wgi/index.aspx#home |
| Control of Corruption Index | Transparency International (accessed 7^th^ January 2016).  http://www.transparency.org/research/cpi/overview |
| Out-of-pocket expenditures | WHO data (accessed January 6^th^, 2016).  http://apps.who.int/gho/data/node.main.75 |
| Geographical coordinates | Data downloaded from John Luke Gallup website (accessed January 21^th^, 2016).  https://www.pdx.edu/econ/jlgallup/country-geodata |
| Proportion of land suitable for wheat | Data extracted from Easterly W. 2007. Inequality Does Cause Underdevelopment: Insights from a New Instrument. Journal of Development Economics 2007; **84**: 755–76 |
| Natural disasters | Data on the number of deaths and people affected by natural disasters are taken from EM-DAT: The OFDA/CRED International Disaster Database – [www.emdat.be](http://www.emdat.be/) – Université Catholique de Louvain – Brussels – Belgium. |
| Fragmentalization measures & geographical variables | Alesina A, Michalopoulos S, and Papaioannou E. 2016. “Ethnic Inequality.” Journal of Political Economy 124 (2): 428-488  We use the following measures (listed with the original sources):  Ethnolinguistic fractionalization index. (Desmet et al., 2012)  Esteban and Ray polarization index (controls for distances). (Desmet et al., 2012)  Natural Logarithm of Absolute Latitude from the Equator  Average annual temp (in K)  Terrain Ruggedness Index, (100 m.). (Nunn and Puga (2012))  Fertile soil. (Nunn and Puga (2012))  Tropical Climate. (Nunn and Puga (2012))  Average distance to nearest ice-free coast (1000 km.) (Nunn and Puga (2012)) |
| Population characteristics | From the World Bank’s World Development Indicator dataset we obtained data on the population size, population density, urban share, and the share of migrants in the population. (accessed 4^th^ October 2016) |
| Intensity of conflict | From the World Bank’s World Development Indicator dataset we obtained data on the number of deaths in conflicts. This data is originally published by the Uppsala Conflict Data Program (accessed 4^th^ October 2016)  http://data.worldbank.org/indicator/VC.BTL.DETH |

|  |  | (1) | (2) | (3) | (4) | (5) | (6) | (7) |
| --- | --- | --- | --- | --- | --- | --- | --- | --- |
| (1) | Group I DALYs lost (log), WHO 2012 | 1 |  |  |  |  |  |  |
| (2) | Group 2 DALYs lost (log), WHO 2012 | 0.76 | 1 |  |  |  |  |  |
| (3) | Group 3 DALYs lost (log), WHO 2012 | 0.81 | 0.75 | 1 |  |  |  |  |
| (4) | Group I DALYs lost (log), WHO 2000 | 0.99 | 0.76 | 0.80 | 1 |  |  |  |
| (5) | Group I DALYs lost (log), IHME 2013 | 0.99 | 0.73 | 0.79 | 0.98 | 1 |  |  |
| (6) | Under-five mortality | 0.97 | 0.80 | 0.81 | 0.96 | 0.96 | 1 |  |
| (7) | Maternal mortality | 0.92 | 0.80 | 0.78 | 0.92 | 0.90 | 0.98 | 1 |

*Correlation matrix for dependent variables*

|  |  | (1) | (2) | (3) | (4) | (5) | (6) | (7) | (8) | (9) | (10) | (11) | (12) |
| --- | --- | --- | --- | --- | --- | --- | --- | --- | --- | --- | --- | --- | --- |
| (1) | Group I DALYs lost (log) | 1 |  |  |  |  |  |  |  |  |  |  |  |
| (2) | GNI per capita (log) | -0.82 | 1 |  |  |  |  |  |  |  |  |  |  |
| (3) | Government Effectiveness | -0.64 | 0.71 | 1 |  |  |  |  |  |  |  |  |  |
| (4) | Epidemiological surroundings | 0.93 | -0.73 | -0.51 | 1 |  |  |  |  |  |  |  |  |
| (5) | Poverty gap (1.9) | 0.72 | -0.76 | -0.52 | 0.66 | 1 |  |  |  |  |  |  |  |
| (6) | Poverty gap (3.1) | 0.82 | -0.85 | -0.57 | 0.76 | 0.97 | 1 |  |  |  |  |  |  |
| (7) | Poverty gap (5) | 0.87 | -0.89 | -0.61 | 0.81 | 0.89 | 0.98 | 1 |  |  |  |  |  |
| (8) | Poverty gap (7.5) | 0.89 | -0.91 | -0.64 | 0.82 | 0.82 | 0.93 | 0.99 | 1 |  |  |  |  |
| (9) | Poverty gap (10) | 0.89 | -0.90 | -0.65 | 0.82 | 0.77 | 0.89 | 0.96 | 0.99 | 1 |  |  |  |
| (10) | Poverty gap (10.89) | 0.89 | -0.90 | -0.65 | 0.82 | 0.76 | 0.88 | 0.95 | 0.99 | 1 | 1 |  |  |
| (11) | Poverty gap (12.5) | 0.88 | -0.90 | -0.66 | 0.81 | 0.74 | 0.86 | 0.94 | 0.98 | 1.00 | 0.999 | 1 |  |
| (12) | Poverty gap (15) | 0.87 | -0.89 | -0.67 | 0.80 | 0.71 | 0.83 | 0.92 | 0.97 | 0.99 | 0.99 | 0.998 | 1 |

*Correlation matrix main model*

| Variables | Obs | Mean | Std. Dev. | Min | Max |
| --- | --- | --- | --- | --- | --- |
| Group I DALYs lost, WHO 2012 | 172 | 14429 | 16951 | 713 | 70714 |
| Group 2 DALYs lost, WHO 2012 | 172 | 22863 | 5252 | 11555 | 38411 |
| Group 3 DALYs lost, WHO 2012 | 172 | 4353 | 2289 | 1159 | 18327 |
| Group I DALYs lost, WHO 2000 | 172 | 22522 | 26981 | 957 | 112249 |
| Group I DALYs lost, IHME 2013 | 188 | 13077 | 16552 | 828 | 66868 |
| Under-five mortality | 195 | 35.4 | 36.2 | 2.1 | 172.2 |
| Maternal mortality | 195 | 14.7 | 11.9 | 0.8 | 51.1 |
| GNI per capita | 146 | 17984 | 20065 | 604 | 124506 |
| Government Effectiveness | 210 | 0.00 | 1.00 | -2.23 | 2.21 |
| Control of Corruption (WB) | 210 | 0.00 | 1.00 | -1.59 | 2.39 |
| CPI 2012 Score | 176 | 43.27 | 19.67 | 8 | 90 |
| Out-of-Pocket expenditures (%) | 191 | 31.4 | 18.4 | 0.1 | 74.5 |
| Mean Year of Schooling | 146 | 8.3 | 2.8 | 1.9 | 13.2 |
| Fertility rate | 200 | 2.8 | 1.4 | 1.2 | 7.6 |
| Poverty gap (1.9) | 126 | 6.7 | 9.3 | 0.0 | 40.8 |
| Poverty gap (3.1) | 126 | 13.9 | 15.4 | 0.0 | 59.4 |
| Poverty gap (5) | 126 | 24.1 | 21.2 | 0.0 | 73.2 |
| Poverty gap (7.5) | 126 | 34.4 | 24.6 | 0.1 | 81.7 |
| Poverty gap (10) | 126 | 42.4 | 25.6 | 0.4 | 86.1 |
| Poverty gap (10.89) | 126 | 44.9 | 25.6 | 0.6 | 87.2 |
| Poverty gap (12.5) | 126 | 48.8 | 25.5 | 1.0 | 88.8 |
| Poverty gap (15) | 126 | 54.0 | 24.8 | 1.9 | 90.6 |

*Descriptive statistics*

|  | Autocorrelation 2012-2000 |
| --- | --- |
| Group I DALYs lost, WHO 2012 | 0.95 |
| GNI per capita | 0.97 |
| Government Effectiveness | 0.94 |
| Poverty gap (1.9) | 0.82 |
| Poverty gap (3.1) | 0.86 |
| Poverty gap (5) | 0.91 |
| Poverty gap (7.5) | 0.91 |
| Poverty gap (10) | 0.91 |
| Poverty gap (10.89) | 0.90 |
| Poverty gap (12.5) | 0.91 |
| Poverty gap (15) | 0.90 |

*Autocorrelation in variables of interest between 2012 and 2000*

**Appendix 3 – Robustness checks**

|  | (1) | (2) | (3) | (4) | (5) | (6) | (7) | (8) |
| --- | --- | --- | --- | --- | --- | --- | --- | --- |
|  | Dependent variable: Group I DALYs lost per 100,000 (log) | | | | | | | |
| Poverty line | Maximum Likelihood Estimation | | | | | | | |
| *(in international $)* | 1.9 | 3.1 | 5 | 7.5 | 10 | 10.89 | 12.5 | 15 |
|  |  |  |  |  |  |  |  |  |
| GNI per capita (log) | -0.168*** | -0.112* | -0.0280 | 0.00462 | -0.0170 | -0.0280 | -0.0469 | -0.0702 |
|  | (0.0591) | (0.0649) | (0.0700) | (0.0715) | (0.0700) | (0.0692) | (0.0678) | (0.0658) |
|  |  |  |  |  |  |  |  |  |
| Poverty gap | 0.0807* | 0.153*** | 0.270*** | 0.325*** | 0.304*** | 0.291*** | 0.268*** | 0.240*** |
| *(see poverty line 1st row)* | (0.0459) | (0.0583) | (0.0713) | (0.0773) | (0.0762) | (0.0752) | (0.0732) | (0.0703) |
|  |  |  |  |  |  |  |  |  |
| Government | -0.150*** | -0.155*** | -0.156*** | -0.147*** | -0.138*** | -0.136*** | -0.132*** | -0.127*** |
| effectiveness (WB) | (0.0413) | (0.0408) | (0.0397) | (0.0391) | (0.0395) | (0.0397) | (0.0400) | (0.0405) |
|  |  |  |  |  |  |  |  |  |
| Lambda | 0.691*** | 0.668*** | 0.628*** | 0.607*** | 0.614*** | 0.618*** | 0.626*** | 0.637*** |
|  | (0.0431) | (0.0447) | (0.0469) | (0.0482) | (0.0482) | (0.0480) | (0.0476) | (0.0469) |
|  |  |  |  |  |  |  |  |  |
| Sigma2 | 0.0832*** | 0.0809*** | 0.0765*** | 0.0747*** | 0.0758*** | 0.0763*** | 0.0772*** | 0.0782*** |
|  | (0.0119) | (0.0116) | (0.0109) | (0.0107) | (0.0108) | (0.0109) | (0.0110) | (0.0112) |
|  |  |  |  |  |  |  |  |  |
| Constant | -0.0107 | -0.0103 | -0.00968 | -0.00935 | -0.00946 | -0.00953 | -0.00965 | -0.00982 |
|  | (0.0290) | (0.0286) | (0.0278) | (0.0275) | (0.0277) | (0.0278) | (0.0279) | (0.0281) |
| Observations | 99 | 99 | 99 | 99 | 99 | 99 | 99 | 99 |
| Chi2 | 78.3 | 83.25 | 93.36 | 97.9 | 95.24 | 93.9 | 91.8 | 89.45 |
| Standard errors in parentheses. * p<0.10, ** p<0.05, *** p<0.01. The sample includes 99 countries for which all data is available. All variables are standardized. | | | | | | | | |

|  | (1) | (2) | (3) | (4) | (5) | (6) | (7) | (8) |
| --- | --- | --- | --- | --- | --- | --- | --- | --- |
|  | Dependent variable: Group I DALYs lost per 100,000 (log) | | | | | | | |
| Poverty line | GS-2SLS – weighting matrix identifying direct neighboring countries | | | | | | | |
| *(in international $)* | 1.9 | 3.1 | 5 | 7.5 | 10 | 10.89 | 12.5 | 15 |
|  |  |  |  |  |  |  |  |  |
| GNI per capita (log) | -0.179** | -0.108 | 0.00393 | 0.0374 | 0.00619 | -0.00820 | -0.0328 | -0.0636 |
|  | (0.0710) | (0.0762) | (0.0806) | (0.0810) | (0.0791) | (0.0783) | (0.0770) | (0.0752) |
|  |  |  |  |  |  |  |  |  |
| Poverty gap | 0.117** | 0.207*** | 0.334*** | 0.378*** | 0.354*** | 0.340*** | 0.314*** | 0.280*** |
| *(see poverty line 1st row)* | (0.0551) | (0.0723) | (0.0870) | (0.0919) | (0.0909) | (0.0900) | (0.0881) | (0.0853) |
|  |  |  |  |  |  |  |  |  |
| Government | -0.172*** | -0.179*** | -0.179*** | -0.167*** | -0.156*** | -0.152*** | -0.147*** | -0.141*** |
| effectiveness (WB) | (0.0480) | (0.0473) | (0.0451) | (0.0442) | (0.0449) | (0.0453) | (0.0459) | (0.0468) |
|  |  |  |  |  |  |  |  |  |
| DALYs-C spatial lag (log) | 0.629*** | 0.598*** | 0.584*** | 0.583*** | 0.583*** | 0.585*** | 0.590*** | 0.597*** |
|  | (0.0615) | (0.0634) | (0.0618) | (0.0618) | (0.0634) | (0.0638) | (0.0644) | (0.0648) |
|  |  |  |  |  |  |  |  |  |
| Constant | -0.00325 | -0.00168 | 0.000791 | 0.00162 | 0.00112 | 0.000855 | 0.000399 | -0.000187 |
|  | (0.0340) | (0.0334) | (0.0320) | (0.0314) | (0.0318) | (0.0319) | (0.0323) | (0.0326) |
|  |  |  |  |  |  |  |  |  |
| Observations | 91 | 91 | 91 | 91 | 91 | 91 | 91 | 91 |
| Standard errors in parentheses. * p<0.10, ** p<0.05, *** p<0.01. The sample includes 91 countries for which all data is available. All variables are standardized. | | | | | | | | |

**Appendix 4 – robustness checks**

Controlling for geographical variables and for geographical variables in neighboring countries

Data sources are detailed in Appendix 2. In every odd column, we include a new geographical variable as control. In even column, both the new geographical variable and its average value in surrounding countries are included in the regression. We find no strong and robust effect of these geographical variables. Overall, the coefficients associated with our main variables of interest are not significantly affected by the inclusion of these supplementary controls.

|  | (1) | (2) | (3) | (4) | (5) | (6) | (7) | (8) | (9) | (10) | (11) | (12) |
| --- | --- | --- | --- | --- | --- | --- | --- | --- | --- | --- | --- | --- |
|  | Dependent variable: Group I DALYs lost per 100,000 (log) | | | | | | | | | | | |
|  |  |  |  |  |  |  |  |  |  |  |  |  |
|  |  |  |  |  |  |  |  |  |  |  |  |  |
| GNI per capita (log) | -0.0349 | -0.0398 | -0.0334 | -0.00504 | -0.00818 | -0.0143 | -0.0326 | -0.0276 | -0.0335 | -0.0330 | -0.0340 | -0.0400 |
|  | (0.0708) | (0.0716) | (0.0705) | (0.0732) | (0.0712) | (0.0706) | (0.0704) | (0.0699) | (0.0702) | (0.0702) | (0.0705) | (0.0712) |
|  |  |  |  |  |  |  |  |  |  |  |  |  |
| Poverty gap | 0.248*** | 0.247*** | 0.243*** | 0.269*** | 0.260*** | 0.264*** | 0.248*** | 0.247*** | 0.243*** | 0.244*** | 0.246*** | 0.237*** |
| *(10.89 international-$)* | (0.0784) | (0.0784) | (0.0790) | (0.0814) | (0.0776) | (0.0769) | (0.0786) | (0.0777) | (0.0788) | (0.0787) | (0.0784) | (0.0787) |
|  |  |  |  |  |  |  |  |  |  |  |  |  |
| Government | -0.212*** | -0.212*** | -0.213*** | -0.227*** | -0.249*** | -0.238*** | -0.215*** | -0.213*** | -0.227*** | -0.225*** | -0.213*** | -0.207*** |
| effectiveness (WB) | (0.0616) | (0.0615) | (0.0620) | (0.0623) | (0.0639) | (0.0638) | (0.0620) | (0.0614) | (0.0628) | (0.0629) | (0.0612) | (0.0623) |
|  |  |  |  |  |  |  |  |  |  |  |  |  |
| Absolute latitude | -0.0111 | -0.0322 |  |  |  |  |  |  |  |  |  |  |
|  | (0.0365) | (0.0611) |  |  |  |  |  |  |  |  |  |  |
|  |  |  |  |  |  |  |  |  |  |  |  |  |
| Terrain ruggedness |  |  | -0.00716 | 0.00808 |  |  |  |  |  |  |  |  |
|  |  |  | (0.0299) | (0.0318) |  |  |  |  |  |  |  |  |
|  |  |  |  |  |  |  |  |  |  |  |  |  |
| Soil quality |  |  |  |  | 0.0517 | 0.0708** |  |  |  |  |  |  |
|  |  |  |  |  | (0.0326) | (0.0350) |  |  |  |  |  |  |
|  |  |  |  |  |  |  |  |  |  |  |  |  |
| Tropical climate |  |  |  |  |  |  | 0.000759 | 0.0663 |  |  |  |  |
|  |  |  |  |  |  |  | (0.0324) | (0.0581) |  |  |  |  |
|  |  |  |  |  |  |  |  |  |  |  |  |  |
| Distance to coast |  |  |  |  |  |  |  |  | -0.0222 | -0.00556 |  |  |
|  |  |  |  |  |  |  |  |  | (0.0300) | (0.0562) |  |  |
|  |  |  |  |  |  |  |  |  |  |  |  |  |
| Average temperature |  |  |  |  |  |  |  |  |  |  | 0.0131 | 0.0462 |
|  |  |  |  |  |  |  |  |  |  |  | (0.0368) | (0.0701) |
|  |  |  |  |  |  |  |  |  |  |  |  |  |
| Geography in neighboring |  | 0.0295 |  | -0.0425 |  | -0.0536 |  | -0.0828 |  | -0.0195 |  | -0.0426 |
| countries |  | (0.0730) |  | (0.0330) |  | (0.0384) |  | (0.0608) |  | (0.0560) |  | (0.0741) |
|  |  |  |  |  |  |  |  |  |  |  |  |  |
| Epidemiological | 0.658*** | 0.664*** | 0.673*** | 0.659*** | 0.681*** | 0.652*** | 0.665*** | 0.688*** | 0.670*** | 0.671*** | 0.659*** | 0.674*** |
| surroundings | (0.0612) | (0.0646) | (0.0554) | (0.0563) | (0.0561) | (0.0595) | (0.0565) | (0.0575) | (0.0549) | (0.0550) | (0.0588) | (0.0612) |
|  |  |  |  |  |  |  |  |  |  |  |  |  |
| Constant | -0.0810** | -0.0810** | -0.0815** | -0.0856** | -0.0933*** | -0.0892** | -0.0819** | -0.0817** | -0.0859** | -0.0853** | -0.0812** | -0.0793** |
|  | (0.0348) | (0.0347) | (0.0348) | (0.0346) | (0.0349) | (0.0347) | (0.0348) | (0.0345) | (0.0349) | (0.0349) | (0.0347) | (0.0348) |
|  |  |  |  |  |  |  |  |  |  |  |  |  |
| Observations | 97 | 97 | 97 | 97 | 97 | 97 | 97 | 97 | 97 | 97 | 97 | 97 |
| Standard errors in parentheses. * p<0.10, ** p<0.05, *** p<0.01. All variables are standardized. | | | | | | | | |  |  |  |  |

Controlling for population variables and population variables in neighboring countries

Data sources are detailed in Appendix 2. In every odd column, we include a new variable related to population characteristics. In even column, both the new variable and its average value in surrounding countries are included in the regression. Only ethnolinguistic fragmentation seems to have a significant impact on disease burden. In line with expectations, an increase in ethnolinguistic fragmentation increases the predicted burden of disease. Overall, the coefficients associated with our main variables of interest are not significantly affected by the inclusion of these supplementary controls.

|  | (1) | (2) | (3) | (4) | (5) | (6) | (7) | (8) | (9) | (10) | (11) | (12) |
| --- | --- | --- | --- | --- | --- | --- | --- | --- | --- | --- | --- | --- |
|  | Dependent variable: Group I DALYs lost per 100,000 (log) | | | | | | | | | | | |
|  |  |  |  |  |  |  |  |  |  |  |  |  |
|  |  |  |  |  |  |  |  |  |  |  |  |  |
| GNI per capita (log) | -0.0415 | -0.0406 | -0.0383 | -0.00967 | -0.0137 | -0.00358 | -0.0257 | -0.0264 | -0.0275 | -0.0276 | -0.00625 | 0.00238 |
|  | (0.0678) | (0.0675) | (0.0701) | (0.0706) | (0.0736) | (0.0717) | (0.0688) | (0.0687) | (0.0691) | (0.0690) | (0.0698) | (0.0689) |
|  |  |  |  |  |  |  |  |  |  |  |  |  |
| Poverty gap | 0.252*** | 0.261*** | 0.258*** | 0.285*** | 0.237*** | 0.250*** | 0.257*** | 0.262*** | 0.256*** | 0.251*** | 0.279*** | 0.269*** |
| *(10.89 international-$)* | (0.0756) | (0.0762) | (0.0781) | (0.0781) | (0.0774) | (0.0755) | (0.0793) | (0.0794) | (0.0779) | (0.0785) | (0.0774) | (0.0764) |
|  |  |  |  |  |  |  |  |  |  |  |  |  |
| Government | -0.209*** | -0.207*** | -0.206*** | -0.228*** | -0.217*** | -0.206*** | -0.208*** | -0.205*** | -0.210*** | -0.209*** | -0.222*** | -0.225*** |
| effectiveness (WB) | (0.0586) | (0.0584) | (0.0609) | (0.0610) | (0.0611) | (0.0596) | (0.0600) | (0.0600) | (0.0600) | (0.0599) | (0.0600) | (0.0591) |
|  |  |  |  |  |  |  |  |  |  |  |  |  |
| Ethnolinguistic fragmentation | 0.0998*** | 0.110*** |  |  |  |  |  |  |  |  |  |  |
|  | (0.0344) | (0.0367) |  |  |  |  |  |  |  |  |  |  |
|  |  |  |  |  |  |  |  |  |  |  |  |  |
| Ethnolinguistic polarization |  |  | 0.0348 | 0.0426 |  |  |  |  |  |  |  |  |
|  |  |  | (0.0294) | (0.0292) |  |  |  |  |  |  |  |  |
|  |  |  |  |  |  |  |  |  |  |  |  |  |
| Urban share |  |  |  |  | -0.0208 | -0.0671 |  |  |  |  |  |  |
|  |  |  |  |  | (0.0396) | (0.0425) |  |  |  |  |  |  |
|  |  |  |  |  |  |  |  |  |  |  |  |  |
| Population density |  |  |  |  |  |  | -0.0209 | -0.0169 |  |  |  |  |
|  |  |  |  |  |  |  | (0.0292) | (0.0298) |  |  |  |  |
|  |  |  |  |  |  |  |  |  |  |  |  |  |
| Migrant share |  |  |  |  |  |  |  |  | 0.000827 | -0.000701 |  |  |
|  |  |  |  |  |  |  |  |  | (0.0295) | (0.0296) |  |  |
|  |  |  |  |  |  |  |  |  |  |  |  |  |
| Population (log) |  |  |  |  |  |  |  |  |  |  | -0.0423 | -0.0451 |
|  |  |  |  |  |  |  |  |  |  |  | (0.0289) | (0.0285) |
|  |  |  |  |  |  |  |  |  |  |  |  |  |
| Control var. in neighboring |  | -0.0375 |  | -0.0614* |  | 0.115** |  | -0.0194 |  | 0.0153 |  | 0.0525* |
| Countries |  | (0.0500) |  | (0.0329) |  | (0.0447) |  | (0.0292) |  | (0.0336) |  | (0.0298) |
|  |  |  |  |  |  |  |  |  |  |  |  |  |
| Epidemiological | 0.589*** | 0.608*** | 0.662*** | 0.624*** | 0.681*** | 0.743*** | 0.670*** | 0.667*** | 0.662*** | 0.675*** | 0.661*** | 0.656*** |
| surroundings | (0.0573) | (0.0628) | (0.0543) | (0.0571) | (0.0521) | (0.0560) | (0.0540) | (0.0538) | (0.0535) | (0.0602) | (0.0530) | (0.0524) |
|  |  |  |  |  |  |  |  |  |  |  |  |  |
| Constant | -0.0785** | -0.0783** | -0.0789** | -0.0854** | -0.0795** | -0.0771** | -0.0763** | -0.0754** | -0.0768** | -0.0769** | -0.0807** | -0.0816** |
|  | (0.0333) | (0.0332) | (0.0344) | (0.0340) | (0.0338) | (0.0329) | (0.0336) | (0.0335) | (0.0336) | (0.0336) | (0.0334) | (0.0329) |
|  |  |  |  |  |  |  |  |  |  |  |  |  |
| Observations | 97 | 97 | 97 | 97 | 99 | 99 | 99 | 99 | 99 | 99 | 99 | 99 |
| Standard errors in parentheses. * p<0.10, ** p<0.05, *** p<0.01. All variables are standardized. | | | | | | | | |  |  |  |  |

Controlling for natural disasters and conflicts and for these variables in neighboring countries

Data sources are detailed in Appendix 2. In every odd column, we include a new variable as control. In even column, both the new variable and its average value in surrounding countries are included in the regression. The coefficient associated with the number of people affected by natural disasters surprisingly negative, but low. Because of their vulnerability, countries subject to natural disaster may have invested in better health systems, either because it is a priority for them of because they benefited from more international aid. Overall, the coefficients associated with our main variables of interest are not significantly affected by the inclusion of these supplementary controls.

|  | (1) | (2) | (3) | (4) | (5) | (6) |
| --- | --- | --- | --- | --- | --- | --- |
|  | Dependent variable: Group I DALYs lost per 100,000 (log) | | | | | |
|  |  |  |  |  |  |  |
|  |  |  |  |  |  |  |
| GNI per capita (log) | -0.0115 | -0.0171 | -0.0302 | -0.0315 | -0.0309 | -0.0301 |
|  | (0.0681) | (0.0672) | (0.0694) | (0.0692) | (0.0693) | (0.0692) |
|  |  |  |  |  |  |  |
| Poverty gap | 0.307*** | 0.313*** | 0.256*** | 0.250*** | 0.249*** | 0.244*** |
| *(10.89 international-$)* | (0.0787) | (0.0776) | (0.0779) | (0.0778) | (0.0779) | (0.0776) |
|  |  |  |  |  |  |  |
| Government | -0.220*** | -0.219*** | -0.208*** | -0.208*** | -0.205*** | -0.204*** |
| effectiveness (WB) | (0.0595) | (0.0587) | (0.0602) | (0.0600) | (0.0605) | (0.0606) |
|  |  |  |  |  |  |  |
| Number of people affected | -0.0661** | -0.0966*** |  |  |  |  |
| by natural disasters (log) | (0.0315) | (0.0356) |  |  |  |  |
|  |  |  |  |  |  |  |
| Number of death |  |  | 0.00972 | 0.00535 |  |  |
| due to natural disasters (log) |  |  | (0.0298) | (0.0302) |  |  |
|  |  |  |  |  |  |  |
| Number of death |  |  |  |  | 0.0149 | 0.00992 |
| due to violent conflict (log) |  |  |  |  | (0.0301) | (0.0313) |
|  |  |  |  |  |  |  |
| Control var. in neighboring |  | 0.0663* |  | 0.0275 |  | 0.0192 |
| countries |  | (0.0375) |  | (0.0338) |  | (0.0350) |
|  |  |  |  |  |  |  |
| Epidemiological | 0.653*** | 0.617*** | 0.656*** | 0.646*** | 0.663*** | 0.660*** |
| surroundings | (0.0525) | (0.0554) | (0.0541) | (0.0561) | (0.0531) | (0.0546) |
|  |  |  |  |  |  |  |
| Constant | -0.0825** | -0.0816** | -0.0761** | -0.0761** | -0.0755** | -0.0750** |
|  | (0.0337) | (0.0333) | (0.0337) | (0.0336) | (0.0337) | (0.0337) |
|  |  |  |  |  |  |  |
| Observations | 98 | 98 | 99 | 99 | 99 | 99 |
| Standard errors in parentheses. * p<0.10, ** p<0.05, *** p<0.01. All variables are standardized. | | | | | | |

Controlling for GNI per capita (log), poverty gap and government effectiveness in neighboring countries

Column (1) reproduces benchmark results. In columns (2) to (4), we observe that GNI per capita (log), poverty gap and government effectiveness in neighboring countries enter significantly in the regression. The sign of these coefficients is unexpected. According to the regression in column (2) for example, an increase in GNI per capita in neighboring countries should on average increase the burden of disease. The effect of the epidemiological surroundings is reinforced by the inclusion of these variables. When the three variables are controlled for, it is GNI per capita (log) in neighboring countries which captures the variation. In columns (6) to (12), we consider OLS regressions to be able to identify multicollinearity problems. In columns (6) to (10), we do not control for the epidemiological surroundings. We observe that the coefficients associated variables of interest in neighboring countries is reversed compared to columns (2) to (4) where the epidemiological surroundings is controlled for. In columns (9) and (10), we further control for the epidemiological surroundings. In column (9), the Variance Inflation Factors (VIF) of the GNI per capita (log) in neighboring countries, of poverty in neighboring countries, and of the epidemiological surroundings are extremely high (22.5, 35.9 and 19.1 respectively), indicating a serious problem of multicollinearity. In column (10), the Variance Inflation Factors (VIF) of the GNI per capita (log) in neighboring countries and the epidemiological surroundings are close to the rule of thumb of 10 signaling serious multicollinearity (10.2 and 9.8 respectively) (O’Brien 2007). We therefore suspect that the weird coefficients of GNI per capita, poverty gap and government effectiveness in neighboring countries in columns (2) to (4) are driven by multicollinearity. It is worth noting that the coefficients associated with GNI per capita (log), poverty gap and government effectiveness are relatively stable across specifications; the only exception is the coefficient of the poverty gap, which capture the effect of the epidemiological surroundings when this latter variable is omitted.

|  | (1) | (2) | (3) | (4) | (5) | (6) | (7) | (8) | (9) | (10) | (11) | (12) |
| --- | --- | --- | --- | --- | --- | --- | --- | --- | --- | --- | --- | --- |
|  | Dependent variable: Group I DALYs lost per 100,000 (log) | | | | | | | | | | | |
|  | GS-2SLS | | | | | OLS | | | | | | |
|  |  |  |  |  |  |  |  |  |  |  |  |  |
| GNI per capita (log) | -0.0274 | -0.122* | -0.0732 | -0.0448 | -0.117 | -0.0326 | 0.106 | 0.0450 | 0.00729 | -0.00852 | -0.101 | -0.105 |
|  | (0.0690) | (0.0718) | (0.0723) | (0.0678) | (0.0721) | (0.116) | (0.0980) | (0.0846) | (0.108) | (0.0870) | (0.0739) | (0.0730) |
|  |  |  |  |  |  |  |  |  |  |  |  |  |
| Poverty gap | 0.256*** | 0.186** | 0.205** | 0.215*** | 0.182** | 0.788*** | 0.543*** | 0.406*** | 0.696*** | 0.359*** | 0.207*** | 0.213*** |
| *(10.89 international-$)* | (0.0775) | (0.0764) | (0.0806) | (0.0775) | (0.0767) | (0.108) | (0.0966) | (0.0886) | (0.102) | (0.0885) | (0.0774) | (0.0766) |
|  |  |  |  |  |  |  |  |  |  |  |  |  |
| Government | -0.210*** | -0.207*** | -0.212*** | -0.237*** | -0.217*** | -0.160 | -0.195** | -0.199*** | -0.117 | -0.213*** | -0.216*** | -0.207*** |
| effectiveness (WB) | (0.0600) | (0.0576) | (0.0600) | (0.0598) | (0.0591) | (0.101) | (0.0833) | (0.0731) | (0.0936) | (0.0735) | (0.0612) | (0.0592) |
|  |  |  |  |  |  |  |  |  |  |  |  |  |
| GNI per capita (log) |  | 0.311*** |  |  | 0.281** |  | -0.443*** |  |  | 0.283* | 0.281** | 0.252*** |
| in neighboring countries |  | (0.0911) |  |  | (0.126) |  | (0.0659) |  |  | (0.157) | (0.131) | (0.0860) |
|  |  |  |  |  |  |  |  |  |  |  |  |  |
| Poverty gap |  |  | -0.337** |  | 0.0182 |  |  | 0.538*** |  | 0.861*** | 0.136 |  |
| in neighboring countries |  |  | (0.160) |  | (0.180) |  |  | (0.0579) |  | (0.145) | (0.165) |  |
|  |  |  |  |  |  |  |  |  |  |  |  |  |
| Gov. effectiveness (WB) |  |  |  | 0.106** | 0.0353 |  |  |  | -0.233*** | 0.0679 | 0.0399 |  |
| in neighboring countries |  |  |  | (0.0467) | (0.0533) |  |  |  | (0.0563) | (0.0660) | (0.0552) |  |
|  |  |  |  |  |  |  |  |  |  |  |  |  |
| Epidemiological | 0.662*** | 0.963*** | 1.023*** | 0.765*** | 0.948*** |  |  |  |  |  | 0.776*** | 0.846*** |
| surroundings | (0.0537) | (0.101) | (0.179) | (0.0686) | (0.155) |  |  |  |  |  | (0.120) | (0.0876) |
|  |  |  |  |  |  |  |  |  |  |  |  |  |
| Constant | -0.0769** | -0.0808** | -0.0833** | -0.0872*** | -0.0835** | -0.0508 | -0.0621 | -0.0633 | -0.0371 | -0.0676* | -0.0687** | -0.0657* |
|  | (0.0336) | (0.0323) | (0.0338) | (0.0332) | (0.0325) | (0.0564) | (0.0467) | (0.0410) | (0.0523) | (0.0404) | (0.0336) | (0.0332) |
|  |  |  |  |  |  |  |  |  |  |  |  |  |
| Observations | 99 | 99 | 99 | 99 | 99 | 99 | 99 | 99 | 99 | 99 | 99 | 99 |
| R² |  |  |  |  |  | 0.793 | 0.860 | 0.892 | 0.825 | 0.899 | 0.931 | 0.930 |
| Standard errors in parentheses. * p<0.10, ** p<0.05, *** p<0.01. All variables are standardized. | | | | | | | | | | | | |
